# Secrecy Performance Analysis of Cognitive Sensor Radio Networks with an EH-Based Eavesdropper

**DOI:** 10.3390/s17051026

**Published:** 2017-05-04

**Authors:** Aiwei Sun, Tao Liang, Bolun Li

**Affiliations:** 1 Institute of Communications Engineering, PLA University of Science and Technology, No. 2 Biaoying, Qinhuai District, Nanjing 210007, China; libolun21@sohu.com; 2 Nanjing Telecommunication Technology Institute, No. 18 Houbiaoying, Qinhuai District, Nanjing 210007, China; liangt61@sina.cn

**Keywords:** physical layer security, cognitive sensor radio networks, secrecy outage performance, Monte Carlo simulations

## Abstract

Security and privacy are crucial for cognitive sensor radio networks (CSRNs) due to the possible eavesdropping between secondary sensors and the secondary fusion center. Motivated by this observation, we investigate the physical layer security performance of CSRNs with an external energy harvesting (EH)-based eavesdropper. Considering the underlay working paradigm of CSRNs, the transmit power of the secondary sensor node must be adjusted to guarantee the quality-of-service (QoS) of the primary user. Hence, two different interference power constraint scenarios are studied in this paper. To give an intuitive insight into the secrecy performance of the considered wiretap scenarios, we have derived the closed-form analytical expressions of secrecy outage probability for both of the considered cases. Monte Carlo simulation results are also performed to verify the theoretical analysis derived, and show the effect of various parameters on the system performance.

## 1. Introduction

Wireless sensor networks (WSNs), which often operate on the unlicensed spectrum (e.g., Industrial, Scientific, Medical (ISM) band), have been widely used in various areas such as environmental monitoring and event detection. However, with the growing proliferation of wireless technologies, the spectrum for WSNs is becoming more and more overcrowded. One promising solution to address this problem is spectrum sharing technologies via cognitive radio (CR). To date, a large number of works have been devoted to the evolution of various aspects of spectrum prediction [1], spectrum sensing [2], and resource management [3]. The above observations provide motivation to study cognitive sensor radio networks (CSRNs) [4,5], which integrate the advantages of CR and WSNs, and have also been considered as an opportunity to realize reliable and low-cost remote monitoring systems.

On the other hand, with the rapid growth of wireless services, energy consumption issues for CSRNs have, in recent years, become increasingly critical, and different energy-efficient optimization algorithms for CSRNs have been investigated [6,7]. However, the sensors are often deployed in remote areas, which makes it inconvenient and infeasible to recharge or replace the batteries frequently. In this situation, energy harvesting (EH) technology has attracted significant interest from industry and the academic community [8,9] because it can effectively alleviate the energy scarcity of WSNs and low-power-consuming equipment. In particular, ambient radio signal can be another safe and convenient energy source since it carries energy and information simultaneously. Consequently, the idea of wireless information and power transfer (WIPT) has been proposed recently, and it has been studied in different communication scenarios (see, e.g., [10,11,12,13,14,15,16,17,18,19,20] and the references therein). To investigate the energy scarcity problem in energy-constraint wireless networks, the authors of [10] firstly proposed a capacity-energy function to characterize the fundamental tradeoffs in WIPT systems. In [11], the authors extended the work in [10] to include frequency-selective single antenna additive white Gaussian noise (AWGN) channels with the average power constraint. In addition, various beam-forming technologies have been proposed in diverse scenarios such as broadcast channels [12,13,14,15,16], relaying channels [17,18], interference channels [19,20], to optimize the transmission performance at the information decoding (ID) users and the harvested energy at the EH users simultaneously.

Moreover, due to the openness of the wireless medium, the dual purposes of energy and information transmission, and the dynamic architecture of the CR system, the wireless information in CSRNs is more susceptible to eavesdropping [21]. Besides, owing to its capability of both information decoding and energy harvesting, the confidential information of secondary transmission can be easily overheard by the EH-based eavesdropper. However, traditional cryptographic techniques will face great challenges, since the eavesdropper can decode the confidential information with the development of the computational ability of a computer. Thus, physical layer security is now emerging as a complementary secure communication method to defend against eavesdroppers, which can effectively enhance the secrecy performance of wireless channels [22,23,24]. Moreover, physical layer security has also been studied in various multi-antenna WIPT systems [25,26,27].

Note that the aforementioned works mainly focus on the aspects of transmission strategy design [28], performance optimization algorithm [29,30], resource management [31,32,33], and few works have investigated the secrecy performance analysis of CSRNs. Different from [23,34], this paper investigates the physical layer security performance of CSRNs with an EH-based enemy fusion center, wherein, due to the PU’s interference temperature constraint, the transmit power of the secondary sensor transmitter (ST) is largely restrained, which has greatly affected the system transmission performance of the secondary sensor network. In addition, the EH-based enemy fusion center has the capability to overhear the confidential message of ST if they do not harvest energy as presumed. In this context, we investigate the impact of an EH-based eavesdropper on the physical layer security performance of the CSRNs. The main contributions of this paper can be summarized as follows:

(1) Considering the interference temperature issues of PU, the closed-form expressions of the secrecy outage probability (SOP) and the average secrecy rate (ASR) are derived, which are validated by Monte Carlo simulations;
(2) Two different scenarios are studied: Case 1, the transmit power of the ST is only affected by the interference power constraint of the PU, and Case 2, the transmit power of the ST is limited by the maximal transmit power of ST itself and the interference power constraint for PU simultaneously;
(3) The effects of various parameters, (such as power splitting factor, link power gain ratio, target secrecy rate), on the physical layer secrecy performance of the CSRNs are investigated, which can give an intuitive insight into the secrecy performance of the considered system.

The remainder of this paper is organized as follows. System model and channel model are introduced in Section 2. In Section 3, we investigate the secrecy performance of the considered system, and derive the closed-form expressions of the secrecy performance metrics for two cases. Numerical results are presented to illustrate the proposed solutions in Section 4. Finally, Section 5 provides some concluding remarks.

## 2. System Model

**Figure 1:**
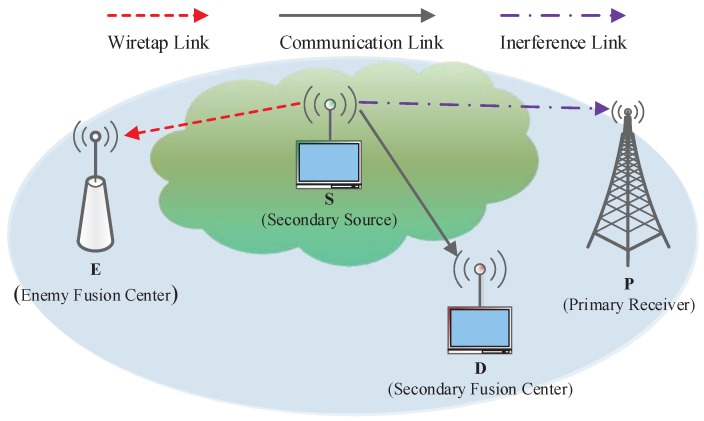
Cognitive sensor radio network system model.

We consider an underlay CSRN as shown in Figure 1, which consists of an unlicensed secondary user (SU) system, a licensed primary user (PU) system, and an external eavesdropper. The SU system consists of a secondary source sensor (S) and a secondary information fusion center (D), which share the same spectrum band with the licensed PU system. Here, we assume that the PU system consists of a single antenna primary receiver (P) as in [35]. The primary transmitter is located far away from the CSRN. Thus, it causes no interference to the SUs. An EH-based enemy fusion center (E) acts as a potential eavesdropper to overhear the SUs’ confidential information. All communication nodes are equipped with one antenna, they also operate in time slot mode for easy implementation. We further assume an independent and quasi-static Rayleigh fading channel model, such that the channel state information remain unchanged during each packet duration, but independently vary from one block to another block. We denote $h_{\mathrm{ab}}$ as the instantaneous link power gain of the link ‘*a*→*b*’, where, *a* denotes the transmitter S, and *b* denotes the corresponding receivers, with $b\in \left\{P,\;D,\;E\right\}$, all link power gains are random variables (RVs) and subject to exponential distribution with parameter, $\lambda_{ab}=1/\Omega_{ab}$, $\Omega_{ab}$ denotes the variances of $h_{ab}$. Specifically, $h_{\mathrm{sp}}$, $h_{\mathrm{sd}}$ and $h_{\mathrm{se}}$ are denoted as the channel gains of the link S→P, S→D and S→E, respectively.

**Figure 2:**
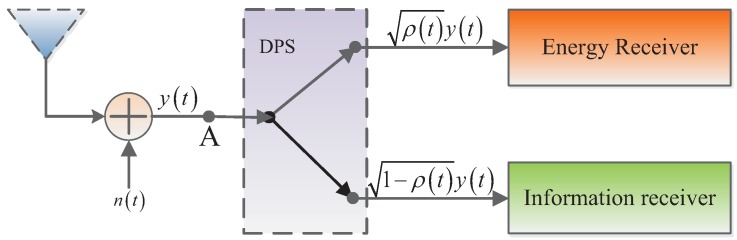
Power splitting architecture of the energy harvesting (EH)-based enemy fusion center.

In this paper, the basic power splitting architecture of the EH receiver is shown in Figure 2. This was initially proposed in [12]. The received radio frequency (RF) signal at the EH receiver is then split into a dynamic power splitter (DPS), no noise is assumed to be induced at the DPS. After the DPS, one part of the received power is used for information decoding, which takes about a $\rho \left(t\right)$ portion of the total received power; the other $1-\rho \left(t\right)$ part is used for energy harvesting. For the eavesdropper, the received signal will be converted to baseband signal after a series of standard operations. As a result, the signal will be corrupted by another noise $n_{p}\left(t\right)$, which is assumed to be AWGN with variance $\sigma_{p}^{2}$.

By denoting x(t) as the data packet transmitted to *Y* at time *t*, with $E\left({\left|x\left(t\right)\right|}^{2}\right)=1$, the signals received at the D and E (potential eavesdropper) can be given as

(1) $$y_{D}=\sqrt{P_{t}\cdot h_{\mathrm{sd}}\left(t\right)}x\left(t\right)+n_{D}\left(t\right),$$

(2) $$y_{E}=\sqrt{\rho \left(t\right)}\sqrt{P_{t}\cdot h_{\mathrm{se}}\left(t\right)}x\left(t\right)+n_{E}\left(t\right)+n_{p}\left(t\right),$$

respectively, where, $P_{t}$ is the transmit power of the S, $n_{D}\left(t\right)$ and $n_{E}\left(t\right)$ are the signal processing noise at the D and E, with noise power $N_{0}$. The time index *t* is ignored below unless necessary in the sequel for notational convenience.

## 3. Secrecy Performance Analysis

In this section, physical layer security issues of the considered cognitive sensor system are investigated, and we concentrate on the performance metrics, including secrecy outage probability and average secrecy rate, which can provide an intuitive insight into the impact of the various system parameters on the transmission security. Then, considering the underlay working mode of the CSRN, two cases are considered. Case 1: we consider that the transmit power of the S is only affected by the interference power constraint of the primary users, and Case 2, we consider that the transmit power of the S is limited by the maximal transmit power of itself and the interference power constraint for P simultaneously, which will be investigated separately in the follow-up work.

### 3.1. Case 1: Interference Power Constraint for the Secondary Transmitter

Considering the interference temperature constraint of the P, the transmit power of S is mainly limited by interference power to the P, which is a common assumption [36]. Thus, the transmit power of the S can be given as

(3) $$P_{t}=\frac{P_{\mathrm{th}}}{h_{\mathrm{sp}}},$$

where, $P_{\mathrm{th}}$ is the predefined interference power threshold, which denotes the maximal interference power that S are allowed to cause to the P. After this, the signal-to-noise ratios (SNRs) at D and E are given as

(4) $$\psi_{D}=\frac{P_{\mathrm{th}}h_{\mathrm{sd}}}{N_{0}h_{\mathrm{sp}}},$$

(5) $$\psi_{E}=\frac{\rho P_{\mathrm{th}}h_{\mathrm{se}}}{\left(\rho N_{0}+\sigma_{p}^{2}\right)h_{\mathrm{sp}}},$$

respectively. By denoting: *u* = $h_{\mathrm{sp}}$, *x* = $\psi_{D}$, *y* = $\psi_{E}$, $\psi_{D}$ and $\psi_{E}$ are also subject to expontional distribution with parameters $\lambda_{\psi_{D}}$ and $\lambda_{\psi_{E}}$, and their probability density functions (PDFs) can be expressed as

(6) $$f_{\psi_{D}}\left(x\right)=\lambda_{\psi_{D}}\exp\left(-\lambda_{\psi_{D}}x\right),$$

(7) $$f_{\psi_{E}}\left(y\right)=\lambda_{\psi_{E}}\exp\left(-\lambda_{\psi_{E}}y\right),$$

where,

(8) $$\lambda_{\psi_{D}}=\frac{\lambda_{\mathrm{sd}}N_{0}u}{P_{\mathrm{th}}},$$

(9) $$\lambda_{\psi_{E}}=\frac{\left(\rho N_{0}+\sigma_{p}^{2}\right)\lambda_{\mathrm{se}}u}{\rho P_{\mathrm{th}}},$$

respectively.

Based on the above analysis, the instantaneous secrecy capacity of secondary transmission link can be further given as follows:

(10) $$\begin{array}{c} C_{\sec}\left(u\right)={\left[\log_{2}\left(1+\psi_{D}\right)-\log_{2}\left(1+\psi_{E}\right)\right]}^{+}, \end{array}$$

here, ${\left[x\right]}^{+}=\max\left\{x,0\right\}$.

#### 3.1.1. Secrecy Outage Probability

As in previous works [22,23,24,25], the SOP is defined as the probability that instantaneous secrecy rate is below a predefined threshold value $R_{s}$.

**Lemma** **1.** *The secrecy outage probability of the considered system under the predefined* $R_{s}$ *for the first case can be calculated as,*

(11) $$\mathrm{SOP}\left(R_{s}\right)=1-\frac{K\lambda_{\mathrm{sp}}P_{\mathrm{th}}}{L+\lambda_{\mathrm{sp}}P_{\mathrm{th}}},$$

*where,*

(12) $$K=\frac{\left(\rho N_{0}+\sigma_{p}^{2}\right)\lambda_{\mathrm{se}}}{\rho N_{0}\lambda_{\mathrm{sd}}2^{R_{s}}+\left(\rho N_{0}+\sigma_{p}^{2}\right)\lambda_{\mathrm{se}}},\;L=N_{0}\lambda_{\mathrm{sd}}\left(2^{R_{s}}-1\right).$$

Based on Equations (11) and (12), the exact SOP value for Case 1 can be calculated under arbitrary predefined target secrecy rate $R_{s}$ and arbitrary interference power threshold $P_{\mathrm{th}}$, which can effectively show the physical layer security performance of the considered system.

**Proof.**  From Equation (10), the SOP conditioned on *u* can be expressed as

(13) $$\begin{array}{cc} \mathrm{SOP}\left(R_{s}\left|u\right.\right) & =\mathrm{Pr}\left(C_{\sec}\left(u\right)<R_{s}\right)\\ & =\mathrm{Pr}\left(\log_{2}\left(\frac{1+\psi_{D}}{1+\psi_{E}}\right)<R_{s}\right)\\ & =\mathrm{Pr}\left(\psi_{D}-\alpha \psi_{E}<\alpha -1\right), \end{array}$$

where, $\mathrm{Pr}\left(\cdot \right)$ denotes the probability of the closed. For notational convenience, we denote, $\alpha =2^{R_{s}}$. Recall from Equation (7), the PDF of $\alpha \psi_{E}$ should be firstly obtained as

(14) $$f_{\alpha \psi_{E}}\left(x\right)=\frac{\lambda_{\psi_{E}}}{\alpha }\exp\left(-\frac{\lambda_{\psi_{E}}}{\alpha }x\right).$$

☐

Further, let us denote $z=\psi_{D}-\alpha \psi_{E}$, combining Equations (6) and (13), the PDF of *z* can be calculated as follows:

(15) $$f_{Z}\left(z\right)=\left\{\begin{array}{c} \int_{0}^{\infty }f_{\psi_{D}}\left(z+x\right)\cdot f_{\alpha \psi_{E}}\left(x\right)dx=A,\;\;\;\;\;z\geq 0\\ \int_{-z}^{\infty }f_{\psi_{D}}\left(z+x\right)\cdot f_{\alpha \psi_{E}}\left(x\right)dx=B,\;\;\;\;\;z<0 \end{array}\right..$$

After some multiplications and transformations, *A* and *B* are easy to be calculated as

(16) $$\begin{array}{c} A=\frac{\lambda_{\psi_{D}}\lambda_{\psi_{E}}}{\alpha \lambda_{\psi_{D}}+\lambda_{\psi_{E}}}\exp\left(-\lambda_{\psi_{D}}z\right), \end{array}$$

(17) $$\begin{array}{c} B=\frac{\lambda_{\psi_{D}}\lambda_{\psi_{E}}}{\lambda_{\psi_{E}}+\alpha \lambda_{\psi_{D}}}\exp\left(\frac{\lambda_{\psi_{E}}z}{\alpha }\right). \end{array}$$

Based on the aforementioned analysis, the $\mathrm{SOP}\left(R_{s}\left|u\right.\right)$ can be further calculated as Equation (18).

(18) $$\begin{array}{c} \begin{array}{cc} \mathrm{SOP}\left(R_{s}\left|u\right.\right) & =\int_{-\infty }^{0}Bdz+\int_{0}^{\alpha -1}Adz\\ & =\int_{-\infty }^{0}\frac{\lambda_{\psi_{D}}\lambda_{\psi_{E}}}{\lambda_{\psi_{E}}+\alpha \lambda_{\psi_{D}}}\exp\left(\frac{\lambda_{\psi_{E}}z}{\alpha }\right)dz\\ & \;+\int_{0}^{\alpha -1}\frac{\lambda_{\psi_{D}}\lambda_{\psi_{E}}}{\alpha \lambda_{\psi_{D}}+\lambda_{\psi_{E}}}\exp\left(-\lambda_{\psi_{D}}z\right)dz\\ & =1-\frac{\lambda_{\psi_{E}}\exp\left(-\lambda_{\psi_{D}}\alpha +\lambda_{\psi_{D}}\right)}{\alpha \lambda_{\psi_{D}}+\lambda_{\psi_{E}}} \end{array} \end{array}$$

Substitute Equations (8) and (9) into Equation (18), the $\mathrm{SOP}\left(R_{s}\left|u\right.\right)$ can also be denoted as

(19) $$\begin{array}{c} \mathrm{SOP}\left(R_{s}\left|u\right.\right)=1-K\exp\left(-\frac{Lu}{P_{\mathrm{th}}}\right), \end{array}$$

where, *K* and *L* have been denoted in Equation (12).

Under PU’s interference temperature constraint, the closed-form expressions for $\mathrm{SOP}\left(R_{s}\right)$ of secondary transmission can be calculated as

(20) $$\mathrm{SOP}\left(R_{s}\right)=\int_{0}^{\infty }\mathrm{SOP}\left(R_{s}\left|u\right.\right)f_{h_{\mathrm{sp}}}\left(u\right)du,$$

where, $f_{h_{\mathrm{sp}}}\left(u\right)$ is the PDF of $h_{\mathrm{sp}}$, with $f_{h_{\mathrm{sp}}}\left(u\right)=\lambda_{\mathrm{sp}}\exp\left(-\lambda_{\mathrm{sp}}u\right)$, the link power gain between S and P, which is also a exponential variable according to previous assumptions [37].

After some multiplications, combining Equation (18) with Equation (20), the $\mathrm{SOP}\left(R_{s}\right)$ for Case 1 can be calculated as

(21) $$\begin{array}{c} \begin{array}{cc} \mathrm{SOP}\left(R_{s}\right) & =\int_{0}^{\infty }\left[1-K\exp\left(-\frac{uL}{P_{\mathrm{th}}}\right)\right]\lambda_{\mathrm{sp}}\exp\left(-\lambda_{\mathrm{sp}}u\right)du\\ & =1-\frac{K\lambda_{\mathrm{sp}}P_{\mathrm{th}}}{L+\lambda_{\mathrm{sp}}P_{\mathrm{th}}} \end{array} \end{array}$$

Thus, we have Equations (11) and (12).

#### 3.1.2. Average Secrecy Rate

As defined in previous works [25,26,27,28,29,30,31,32,33,34], secrecy capacity is the maximum rate at which the destination can decode the packets, while the eavesdropper’s bit error probability of decodes approaches one. Here, we will derive the closed-form expressions of average secrecy rate (ASR) based on the fading characteristic of the Rayleigh fading channel. Under the predefined interference value $P_{\mathrm{th}}$ of P, the ASR can be given as

(22) $$C_{\sec}^{\mathrm{ave}}\left(P_{\mathrm{th}}\right)=\int_{0}^{\infty }C_{\sec}^{\mathrm{ave}}\left(P_{\mathrm{th}}\left|u\right.\right)f_{h_{\mathrm{sp}}}\left(u\right)du,$$

and

(23) $$\begin{array}{c} \begin{array}{cc} C_{\sec}^{\mathrm{ave}}\left(P_{\mathrm{th}}\left|u\right.\right) & =\int_{0}^{\infty }\int_{0}^{\infty }C_{\sec}\left(P_{\mathrm{th}}\left|u\right.\right)\\ & \;\times f_{\psi_{D}}\left(\psi_{D}\right)f_{\psi_{E}}\left(\psi_{E}\right)d_{\psi_{D}}d_{\psi_{E}}, \end{array} \end{array}$$

where, $f_{\psi_{D}}\left(\psi_{D}\right)$, and $f_{\psi_{E}}\left(\psi_{E}\right)$ are the probability density function of $\psi_{D}$, and $\psi_{E}$, respectively, which have been given in Equations (6) and (7).

By using the fact that all channels are assumed to suffer the independent and identical Rayleigh distribution, the ASR can also be calculated as

(24) $$C_{\sec}^{\mathrm{ave}}\left(P_{\mathrm{th}}\left|u\right.\right)=\frac{1}{\ln2}C_{s1}^{\mathrm{ave}}\left(P_{\mathrm{th}}\left|u\right.\right)-\frac{1}{\ln2}C_{s2}^{\mathrm{ave}}\left(P_{\mathrm{th}}\left|u\right.\right)$$

where, $C_{s1}^{\mathrm{ave}}\left(P_{\mathrm{th}}\left|u\right.\right)$ and $C_{s2}^{\mathrm{ave}}\left(P_{\mathrm{th}}\left|u\right.\right)$ can be calculated as Equations (25) and (26), respectively.

(25) $$\begin{array}{c} \begin{array}{cc} C_{s1}^{\mathrm{ave}}\left(P_{\mathrm{th}}\left|u\right.\right) & =\int_{0}^{\infty }\int_{0}^{\psi_{D}}\ln\left(1+\psi_{D}\right)f_{\psi_{D}}\left(\psi_{D}\right)\\ & \;\times f_{\psi_{E}}\left(\psi_{E}\right)d\psi_{D}d\psi_{E}, \end{array} \end{array}$$

(26) $$\begin{array}{c} \begin{array}{cc} C_{s2}^{\mathrm{ave}}\left(P_{\mathrm{th}}\left|u\right.\right) & =\int_{0}^{\infty }\int_{\psi_{E}}^{\infty }\ln\left(1+\psi_{E}\right)f_{\psi_{D}}\left(\psi_{D}\right)\\ & \;\times f_{\psi_{E}}\left(\psi_{E}\right)d_{\psi_{D}}d_{\psi_{E}}, \end{array} \end{array}$$

For easy calculation, we denote: $x=\psi_{D}$, $y=\psi_{E}$, combining with Equations (6) and (7), and by using Equation (27) in [38],

(27) $$\int_{0}^{\infty }e^{-\mu x}\ln\left(1+\beta x\right)dx\;=-\frac{1}{\mu }\exp\left(\frac{\mu }{\beta }\right)\mathrm{Ei}\left(-\frac{\mu }{\beta }\right),$$

the $C_{s1}^{\mathrm{ave}}\left(P_{\mathrm{th}}\left|u\right.\right)$ and $C_{s2}^{\mathrm{ave}}\left(P_{\mathrm{th}}\left|u\right.\right)$ can be further given as follows:

(28) $$\begin{array}{c} \begin{array}{cc} C_{s1}^{\mathrm{ave}}\left(P_{\mathrm{th}}\left|u\right.\right) & =\int_{0}^{\infty }\ln\left(1+x\right)f_{x}\left(x\right)dx\int_{0}^{x}f_{y}\left(y\right)dy\\ & =\frac{\lambda_{\psi_{D}}}{\lambda_{\psi_{D}}+\lambda_{\psi_{E}}}\exp\left(\lambda_{\psi_{D}}+\lambda_{\psi_{E}}\right)\mathrm{Ei}\left(-\lambda_{\psi_{D}}-\lambda_{\psi_{E}}\right)\\ & \;-\exp\left(\lambda_{\psi_{D}}\right)\mathrm{Ei}\left(-\lambda_{\psi_{D}}\right), \end{array} \end{array}$$

(29) $$\begin{array}{c} \begin{array}{cc} C_{s2}^{\mathrm{ave}}\left(P_{\mathrm{th}}\left|u\right.\right) & =\int_{0}^{\infty }\ln\left(1+y\right)f_{y}\left(y\right)dy\int_{y}^{\infty }f_{x}\left(x\right)dx\\ & =-\frac{\lambda_{\psi_{E}}}{\lambda_{\psi_{E}}+\lambda_{\psi_{D}}}\exp\left(\lambda_{\psi_{E}}+\lambda_{\psi_{D}}\right)\;\\ & \;\;\times \mathrm{Ei}\left(-\lambda_{\psi_{E}}-\lambda_{\psi_{D}}\right), \end{array} \end{array}$$

respectively, where, $\mathrm{Ei}\left(x\right)$ is the exponential integral function [38]. Substituting Equations (28) and (29) into Equation (24), the ASR conditioned on *u* can be calculated as

(30) $$\begin{array}{c} \begin{array}{cc} C_{\sec}^{\mathrm{ave}}\left(P_{\mathrm{th}}\left|u\right.\right) & =\frac{1}{\ln2}\exp\left(\lambda_{\phi_{D}}+\lambda_{\phi_{E}}\right)\mathrm{Ei}\left(-\lambda_{\phi_{D}}-\lambda_{\phi_{E}}\right)\\ & -\frac{1}{\ln2}\left(\exp\left(\lambda_{\phi_{D}}\right)\mathrm{Ei}\left(-\lambda_{\phi_{D}}\right)\right). \end{array} \end{array}$$

From Equations (22) and (30), the ASR can be given as Equation (31).

(31) $$\begin{array}{c} \begin{array}{cc} C_{\sec}^{\mathrm{ave}}\left(P_{\mathrm{th}}\right) & =\frac{\lambda_{\mathrm{sp}}}{\ln2}\int_{0}^{\infty }\exp\left(-\lambda_{\mathrm{sp}}u\right)\exp\left(\frac{\rho N_{0}\left(\lambda_{\mathrm{sd}}+\lambda_{\mathrm{se}}\right)+\sigma^{2}\lambda_{\mathrm{se}}}{\rho P_{\mathrm{th}}}u\right)\\ & \;\times \mathrm{Ei}\left(-\frac{\rho N_{0}\left(\lambda_{\mathrm{sd}}+\lambda_{\mathrm{se}}\right)+\sigma^{2}\lambda_{\mathrm{se}}}{\rho P_{\mathrm{th}}}u\right)du\\ & \;-\frac{\lambda_{\mathrm{sp}}}{\ln2}\int_{0}^{\infty }\exp\left(-\lambda_{\mathrm{sp}}u\right)\exp\left(\frac{N_{0}\lambda_{\mathrm{sd}}}{P_{\mathrm{th}}}u\right)\mathrm{Ei}\left(-\frac{N_{0}\lambda_{\mathrm{sd}}}{P_{\mathrm{th}}}u\right)du \end{array} \end{array}$$

Based on the aforementioned analysis, by using the following Equation (32) in [38],

(32) $$\int_{0}^{\infty }\mathrm{Ei}\left(-\beta x\right)\exp\left(-\mu x\right)\mathrm{dx}=-\frac{1}{\mu }\ln\left(1+\frac{\mu }{\beta }\right),$$

and performing some simple mathematical manipulations, we obtain the final closed-form expressions of the ASR for Case 1 as

(33) $$\begin{array}{c} \begin{array}{cc} C_{\sec}^{\mathrm{ave}}\left(P_{\mathrm{th}}\right) & =\frac{\lambda_{\mathrm{sp}}}{\ln2}\int_{0}^{\infty }\exp\left(-\Xi_{1}x\right)\mathrm{Ei}\left(-\Xi_{2}x\right)dx\\ & \;-\frac{\lambda_{\mathrm{sp}}}{\ln2}\int_{0}^{\infty }\exp\left(-\Xi_{3}x\right)\mathrm{Ei}\left(-\Xi_{4}x\right)dx\\ & \;=\frac{\lambda_{\mathrm{sp}}}{\ln2}\left[\frac{1}{\Xi_{3}}\ln\left(1+\frac{\Xi_{3}}{\Xi_{4}}\right)-\frac{1}{\Xi_{1}}\ln\left(1+\frac{\Xi_{1}}{\Xi_{2}}\right)\right] \end{array} \end{array}$$

where,

(34) $$\begin{array}{c} \begin{array}{c} \Xi_{1}=\frac{\lambda_{\mathrm{sp}}\rho P_{\mathrm{th}}-\rho N_{0}\lambda_{\mathrm{se}}-\sigma^{2}\lambda_{\mathrm{se}}-\rho N_{0}\lambda_{\mathrm{sd}}}{\rho P_{\mathrm{th}}},\\ \Xi_{2}=\frac{\rho N_{0}\lambda_{\mathrm{se}}+\sigma^{2}\lambda_{\mathrm{se}}+\rho N_{0}\lambda_{\mathrm{sd}}}{\rho P_{\mathrm{th}}},\\ \Xi_{3}=\frac{\lambda_{\mathrm{sp}}P_{\mathrm{th}}-N_{0}\lambda_{\mathrm{sd}}}{P_{\mathrm{th}}},\\ \Xi_{4}=\frac{N_{0}\lambda_{\mathrm{sd}}}{P_{\mathrm{th}}}. \end{array} \end{array}$$

Based on Equations (33) and (34), the exact ASR value for Case 1 can be easily calculated under arbitrary interference power threshold $P_{\mathrm{th}}$.

### 3.2. Case 2: Maximum Source Power Constraint and Interference Power Constraint for the Secondary Transmitter

In this section, we consider the case that the transmit power of the secondary sensor is limited not only by the interference constraint of the PU system, but also the maximal source power of the S itself. The adoption of this assumption is not intended to complicate the system model, but to address a more practical scenario in wireless communication system. In this case, the transmit power of S can be given as

(35) $$P_{t}=\min\left(P_{\max},\frac{P_{\mathrm{th}}}{h_{\mathrm{sp}}}\right),$$

where, $P_{\max}$ is the allowable power of S, $\min\left(\cdot ,\;\cdot \right)$ denotes the minimum value of the two variables in the parentheses. The SNRs at D and E are given as

(36) ϕD=minPmax,PthPthhsphsphsdN0,

(37) ϕE=ρminPmax,PthPthhsphsphseρN0+σp2,

respectively. By denoting: *v* = $h_{\mathrm{sp}}$, the $\phi_{D}$ and $\phi_{E}$ are also subject to expontional distribution with parameter $\lambda_{\phi_{D}}$ and $\lambda_{\phi_{E}}$, where,

(38) λϕD=N0minPmax,PthPthvvλsd,

(39) λϕE=ρN0+σp2ρminPmax,PthPthvvλse.

Here, we adopt the same calculation procedure as that from Equation (6) to Equation (19). After performing some mathematical manipulations, the $\mathrm{SOP}\left(R_{s}\left|v\right.\right)$ can also be obtained as

(40) SOPRsv=1−Kexp−LminPmax,PthPthvv.

Under PU’s interference temperature constraint, the closed-form expressions for the $\mathrm{SOP}\left(R_{s}\right)$ can be calculated as

(41) $$\mathrm{SOP}\left(R_{s}\right)=\int_{0}^{\infty }\mathrm{SOP}\left(R_{s}\left|v\right.\right)f_{h_{\mathrm{sp}}}\left(v\right)dv,$$

After some calculation, the final closed-form expressions of the SOP for Case 2 can be expressed as Equation (42) at the top of the next page.

(42) SOPRs=∫0PthPmax1−Kexp−LminPmax,PthPthxxλspexp−λspxdx+∫PthPmax+∞1−Kexp−LminPmax,PthPthxxλspexp−λspxdx=1−Kexp−L+λspPthPmax+exp−LPmax−λspKPthL+Pthλspexp−L+λspPthPmax,

Equations (11) and (42) give the analytical expressions of the SOP for two distinct scenarios, which can effectively measure the secrecy performance of the considered system, and show the impact of various parameters on the secrecy performance. In the following sections, we will validate the accuracy of the analytical expressions derived through Monte Carlo simulations.

## 4. Discussions

For a cognitive radio sensor network with EH function for the eavesdropper, two conflicting goals exist: the power of the received signal at the eavesdropper is needs to be large for efficient energy harvesting, but it is also needs to be sufficient to decode more confidential information. The performance boundary of the two goals is mainly determined by the ratio of power splitting, which has a great effect on the secrecy performance of the information transmission. In this subsection, we will investigate the average amount of the harvested power under specified system and channel condition. According to the previous assumptions, the instantaneous harvested power can be calculated as follows:

(43) PEH=1−ρ×Pt×hse=1−ρ×hse×minPmax,PthPthhsphsp.

Let us denote: $x=h_{\mathrm{sp}}$, and $y=h_{\mathrm{se}}$, the average harvested power can be given as

(44) PEHave=∫0∞∫0∞PEH×fhspxfhseydxdy=1−ρ×λseλsp∫0∞xe−λsexdx×∫0∞minPmax,PthPthyye−λspydy.

With the help of the expressions in [38]

(45) $$\int_{1}^{\infty }\frac{e^{-\mu x}}{x}dx=-\mathrm{Ei}\left(-\mu \right).$$

After some multiplications, the final closed-form expressions of average harvested power can be given as

(46) $$\begin{array}{c} \begin{array}{cc} P_{\mathrm{EH}}^{\mathrm{ave}} & =\frac{\left(1-\rho \right)\times P_{\max}}{\lambda_{\mathrm{se}}}\;\left(1-\exp\left(-\frac{\lambda_{\mathrm{sp}}P_{\mathrm{th}}}{P_{\max}}\right)\right)\\ & \;-\frac{\left(1-\rho \right)\times \lambda_{\mathrm{sp}}P_{\mathrm{th}}}{\lambda_{\mathrm{se}}}\mathrm{Ei}\left(-\frac{\lambda_{\mathrm{sp}}P_{\mathrm{th}}}{P_{\max}}\right), \end{array} \end{array}$$

where, $\mathrm{Ei}\left(x\right)$ is the same exponential integral function as in Equation (27).

## 5. Simulation Results and Analysis

We have given system model and channel characteristic description in Section 2. Here, numerical results are presented to highlight the impact of various parameters on secure performance of CSRNs. Recall that all channels experience independent and identical Rayleigh fading. Unless otherwise specified, the relevant simulation parameter can be set as follows: $\Omega_{\mathrm{sp}}=10\;\;\mathrm{dB}$, $\lambda_{\mathrm{sp}}=1/\Omega_{\mathrm{sp}}$, $\Omega_{\mathrm{sd}}=30\;\;\mathrm{dB}$, $\lambda_{\mathrm{sd}}=1/\Omega_{\mathrm{sd}}$, τ=ΩseΩseΩsdΩsd, $N_{0}=1$, $\sigma_{p}^{2}=0.9$. For the EH-based eavesdropper, ρ portion of the total received power is used for information decoding, and the other remaining 1−ρ portion is used for energy harvesting. All communication nodes have a single antenna, and work in time slot mode.

### 5.1. Simulation Results for Case 1

For the case 1, considering the quality-of-service (QoS) of the PU system, the transmit power of the secondary sensor is limited by the interference constraint effect. the analytical curves of the SOP are obtained from Equations (11) and (12).

**Figure 3:**
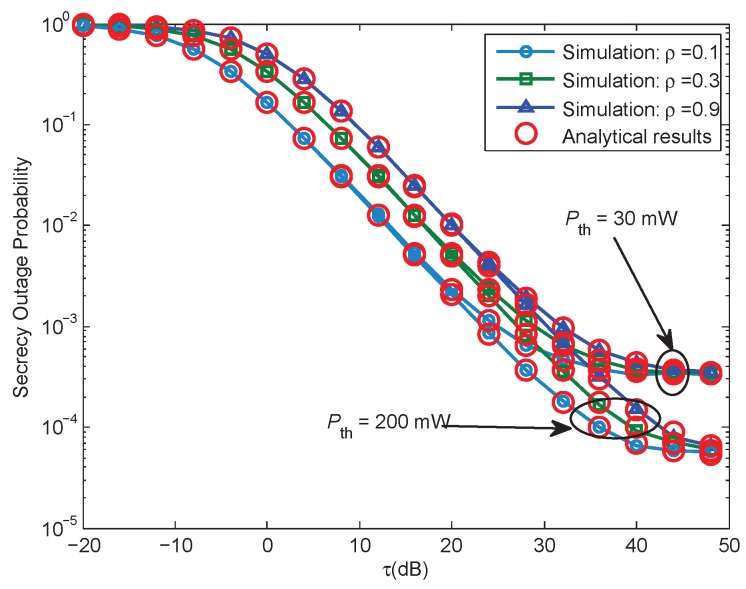
Case 1: Secrecy outage probability versus τ, when $P_{\mathrm{th}}$ = [30, 200] mW, $R_{s}$ = 1 bits/Hz/s, ρ = [0.1, 0.3, 0.9].

Figure 3 gives the SOP performance versus the ratio τ for various power splitting factor ρ, under different interference power threshold $P_{\mathrm{th}}$ for P. For all cases, the derived analytical expressions are in great agreement with the simulation results. We can easily see that: (1) the SOP will decrease with the increase of the τ, which is an expected result, since the main channel has better quality than the wiretap channel; (2) with the same $P_{\mathrm{th}}$ and τ, if we increase the value of ρ, the SOP will be decreased, due to the fact that, higher values of ρ mean more power for the information decoding, which can lead to better secrecy performance; (3) with the same value of τ and ρ, if $P_{\mathrm{th}}$, the interference power threshold is enlarged, and the secrecy performance can be further improved.

**Figure 4:**
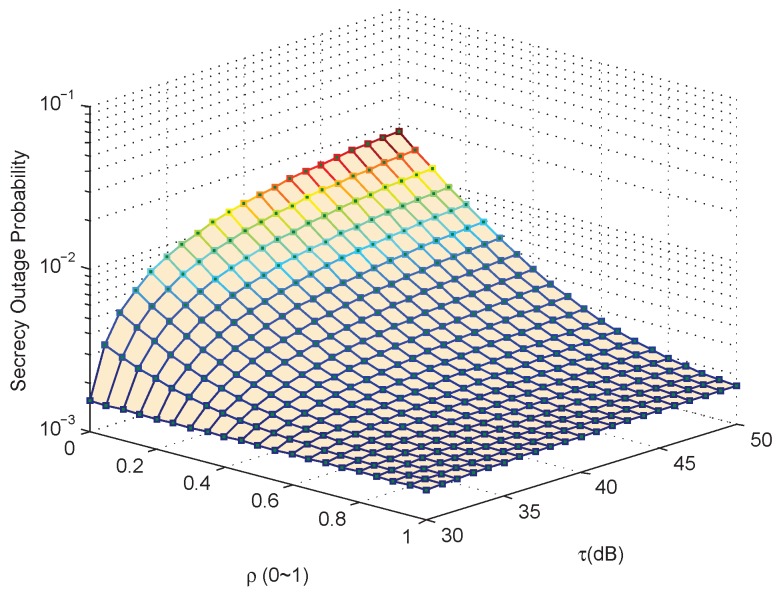
Case 1: Secrecy outage probability versus τ and ρ, when $P_{\mathrm{th}}$ = 200 mW, $R_{s}$ = 5 bits/Hz/s.

Figure 4 gives the SOP performance of the considered system versus τ and various ρ of the EH receiver. For easy implantation, only analytical results are showed in this figure. As can be seen, the SOP apparently decreases with the increase of the EH receiver’s harvested power. These are the expected results since the two goals represent a conflict between the amount of the harvested energy and the rate of the wiretapped information, and they all have a relationship with ρ, the power-splitting factor of the EH-based eavesdropper, if the eavesdropper prefers to harvest more energy through EH function, then less power remains to decode the information, and the secrecy rate of the secondary receiver will decrease, and vice versa.

**Figure 5:**
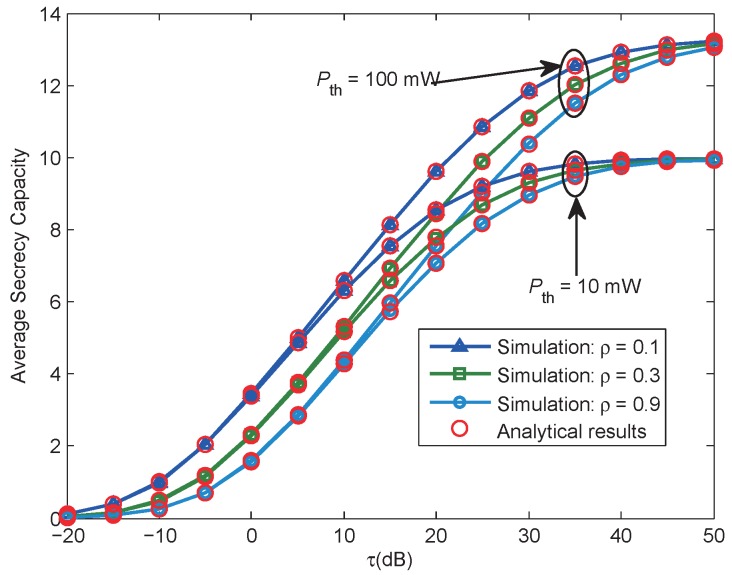
Case 1: Average secrecy rate versus τ, when $P_{\mathrm{th}}$ = 100 mW, $R_{s}$ = 5 bits/Hz/s, ρ = 0.9.

Figure 5 gives the ASR versus the ratio τ for various ρ, under different interference power thresholds, $P_{\mathrm{th}}$ = [10, 100] dBW is provided. For all cases, we can observe that the proposed analytical expressions of ASR given by Equations (33) and (34) are in great agreement with the simulation results, which corroborates the accuracy of the analytical expressions. In addition, the ASR will increase with the increase of the ratio τ, and then converge to a relatively fixed value, since the system performance is limited by the interference power constraint of PR in the high τ region. In addition, as expected, under the same value of τ and ρ, if we enlarge $P_{\mathrm{th}}$, the interference power threshold, the ASR can be further increased.

### 5.2. Simulation Results for Case 2

**Figure 6:**
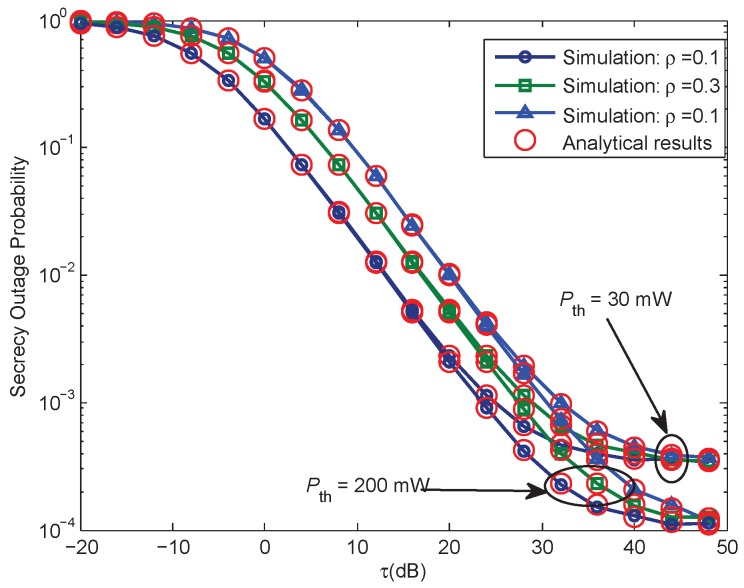
Case 2: Secrecy outage probability versus τ, when $P_{\mathrm{th}}$ = 30 mW, $P_{\max}$ = 10 mW, $R_{s}$ = 1 bits/Hz/s, ρ = 0.9.

In this section, we will consider the second case, the analytical curves of the SOP are obtained from Equation (42), the simulation results are presented as follows:

Figure 6 gives the SOP performance versus the ratio τ for various ρ, ρ=[0.1,0.3,0.9], under different interference power threshold of P, $P_{\mathrm{th}}$ = [10, 30] dB. The curves for both the analytical results and Monte Carlo simulations are presented for Case 2 scenario. We can observe that the secrecy performance curve shown in Figure 6 for Case 2 has a similar trend to that in Figure 3 for Case 1. Under the same parameters set, the main difference is that the secrecy performance for Case 1 performs better than that for Case 2, due to the fact that the SOP for Case 2 is affected by the maximal transmit power constraint of the ST and the interference temperature constraint simultaneously.

**Figure 7:**
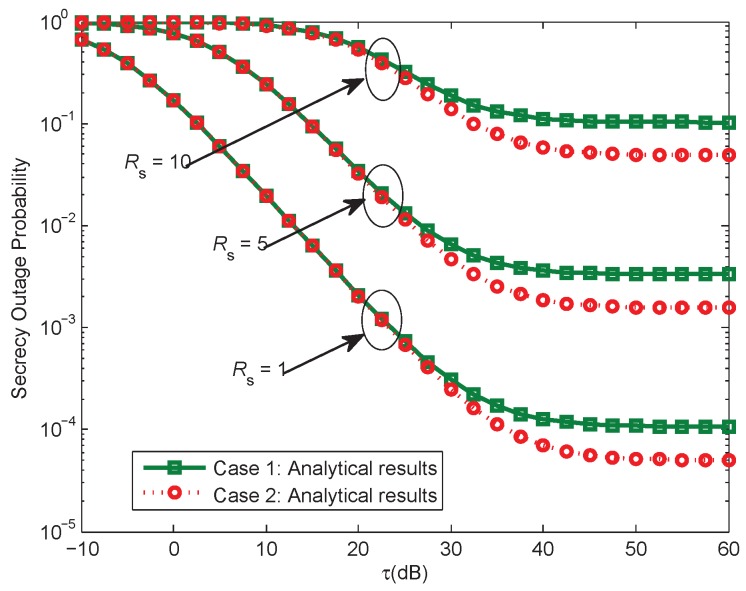
Comparison of secrecy outage probability for the two proposed cases, when when $R_{s}$ = [1, 5, 10] bits/Hz/s, ρ = 0.1, $P_{\mathrm{th}}$ = 200 mW.

Figure 7 provides the SOP versus the ratio τ, under different target secrecy rate $R_{s}$. Only analytical results obtained from Equations (11) and (42) are plotted here, τ ranges from –10 dB to 60 dB. As can be seen, the SOP for case 1 performs better than that for case 2. This is intuitive, since for case 2, the SOP is affected by the maximum power of the secondary source itself additionally, which will somewhat depress the transmit performance.

## 6. Conclusions

This paper has investigated the physical layer security performance of cognitive sensor radio networks. In contrast to conventional security issues, we consider the energy harvesting (EH) function for the eavesdropper. New closed-form expressions of secrecy outage probability for two different cases have been derived, and the impacts of various parameters on secure performance have also been studied. The precise matching between the simulation results and the derived closed-form expressions also validates the theoretical analysis presented in this paper. Furthermore, the proposed analytical models can be readily applied to practical energy harvesting wireless sensor networks design such as power allocation, and transmission policy. As a final remark, this work can be served as an important step for investigating different physical layer security enhancement technologies, e.g., multi-antenna scenarios and full-duplex scenarios, etc., to provide more secrecy transmission methods for cognitive sensor radio networks. Moreover, security in the time switching (TS)-based energy harvesting scheme will be a fundamental and significant research field, which will involve more sophisticated settings and practical considerations in the near future.
